# Tennessee Dental Association

**Published:** 1877-10

**Authors:** 


### TENNESSEE DENTAL ASSOCIATION.

Eleventh annual meeting at Lookout Mountain, June 27th, 1877.

This society convened in its eleventh annual session at Lookout Mountain House, Wednesday afternoon, June 27th, at 2 o'clock.

The society was called to order by its president, H. E. Beach, of Clarksville, and opened with prayer by Dr. Ross, of Nashville. In the absence of the Recording Secretary, Dr. Noel, of Nashville, was elected secretary *pro tem.* The minutes of the previous meeting were read, corrected, and approved.

After the reports of the officers of the society had been heard, together with those of the Executive Committee, and Committee of Arrangements, the business of receiving new members came up.

Dr. Noel suggested the appointment of a committee on membership, and proceeded to make some remarks explanatory of the manner in which the society chanced to hold this meeting in East Tennessee. This was its first meeting in this end of the State. Organized in Memphis, its meetings had been confined to Middle and West Tennessee; for the past six years the meetings had been held in Nashville, because of its central location, and from the unfortunate fact that the West Tennessee profession had lost interest in its welfare. He urged upon the profession of East Tennessee the importance of availing themselves of this opportunity to connect themselves with the society. It had been brought to East Tennessee for their benefit, at great inconvenience to its members, who all, or nearly all, lived in Middle Tennessee. The object of its founders, from the time of its organization, had been to make it strictly a State institution. It was never intended to be narrowed down to any one of our three local sections; nor was the profession in Tennessee numerous enough to support more than one living, thrifty society.

The hours for meeting and adjourning were fixed as follows: Morning session, 10 to 12½; afternoon session, 2½ to 6.

The society was now entertained by the President's Annual Address; the subject being, "The Relation of the Dentist to His Patients."

Drs. Webber and T. E. Beach were appointed a committee to draft an order of business, and, after due consideration, the following subjects were presented for discussion: Dental Education; Anæsthetics and Extraction of Teeth; Operative Dentistry; Dental Prosthesis; Dental Pathology and Therapeutics; Etiology and Prophylaxis.

A resolution was offered by Dr. Ross, to so alter the constitution and by-laws, as to render the recording secretary and treasurer elegible to re-election. Carried.

A paper by Dr. W. H. Thackston, of Farmville, Virginia, on "Dental Education," was now read, and the society proceeded at once to its discussion.

Dr. Ross expressed himself as much entertained with the paper, and thought it ought to be published where every dentist in the State could read it.

Dr. Beach. The tacking of dentistry to medicine has been a hindrance. Dentistry has the same right to the text books as medicine. I concur with Dr. Thackston in the matter of supporting our colleges. Dental Colleges, if properly endowed and supported, could teach anatomy physiology, etc., so fully as to make our students independent of medical schools." The secretary said, "he was very tired of the education wrangle that had been going on between the dental and medical journals since the publication of certain articles in the *Philadelphia Medical Times*, by the editor of that journal. There is no occasion for antagonism between the two professions. We are the legitimate offspring of the mother medicine, and need not be in the least disturbed because a few penny-a-line editors, in the dearth of subject matter for their journals, make a pen tilt at us. We should pay no more heed to it than to the spinning of a thistle down in an Autumn breeze. As far as our rights are concerned, they are always accorded in proportion as deserved, and deserved in proportion as our knowledge rests upon medical lore as a foundation. He liked Dr. Thackston's paper, and believed he did not differ with its author upon any point which it had brought out. He only thought Dr. Thackston had suffered himself to become unnecessarily heated in the little tea-pot tempest, above alluded to.

Dr. Ross. Wish I was a talker, that I might express all I feel upon the subject. I am here impressed with the superiority of the advantages enjoyed by students of the present day, over those possessed when I was a student. Harris, Hayden, Parmly, and such men have passed away; but the journals they established, and the colleges they founded, are still going on with rapidity, extending influence for good. Let students avail themselves of their assistance.

Dr. H. E. Beach. "The paper is from the pen of one who sat, as it were, at the feet of Gamaliel. Dr. Thackston was one of the first students of the Baltimore Dental College. We should establish and sustain schools capable of maintaining a position of dignity with any of the learned professions Dental colleges are not supported, this may be the reason they are deficient.

The discussion was carried on to the hour of adjournment, all participating.

Dr. Noel was appointed to give a clinic to-morrow at 8 o'clock. Adjourned.

### THURSDAY MORNING SESSION.

Discussion of Dr. Thackston's paper continued.

Dr. Lee. I presume I am one of the youngest present. I became a dentist, as it were, by chance. Have not enjoyed the educational advantages that many have had; but do not consider the opportunity passed, and shall do all I can to educate myself and develope with the age.

Dr. Prothro. As President of the East Tennessee Dental Association, I read a paper upon this subject at our last meeting. I took the ground that it is incumbent upon all to sustain our colleges. I am not a graduate of a dental college. Graduated in medicine and practiced that profession sixteen years before commencing the practice of dentistry; twelve years of my life have been devoted to the latter calling. Feel that I should have graduated in a dental college. We cannot make educated dentists in our offices; we may make good office boys of our students, but cannot give them the opportunities afforded at college.

This subject was now passed, and Dr. Noel read a paper upon Anæsthetics.

Dr. Prothro. Though I concur fully with the ideas advanced in the paper, I have found it best in my practice to produce only partial anæsthesia. I use a mixture of chloroform and ether, and only carry it to the extent of slight stupefaction. Formerly gave gas, but found my patients often frightened by the display of apparatus. I use gags and corks, but take the precaution to attach a string to them to prevent accident. It is our duty to examine carefully and diagnose fully the physical condition of the patient, before consenting to administer these agents. We should be provided with a galvanic battery, and everything necessary, in case of emergency, to restore the patient.

Dr. Ross. The question as to what remedies should be applied in case of emergency is an important one, and one we should examine into. Would like to hear the views of the author of the paper upon this point. Would like him to explain why he thinks a partial condition of anæthesia more dangerous than a complete. Once had a case of chloroform that presented very alarming appearances, the patient remaining semi-conscious an hour. Since that time have confined myself to nitrous oxide, using the liquid apparatus of the Johnston Brothers.

Dr. Noel. Those who listened attentively to the paper, will remember that the views advanced in regard to partial anæthesia were taken from Dr. Bartholow's work on *Materia Medica*, and that they are not given as my own, though in the main I subscribe to them. Just why there is less danger in total anæsthesia, is one of those things I don't pretend to know, but the statistics seem to be in favor of that view. I think every dentist who deals largely with anæsthetics should be provided with a powerful galvanic battery, constantly ready for use in case emergent symptoms should arise. That the electric current is near akin to the *vis vitæ*, is witnessed in the powerful throes of muscular contraction which it excites in the dead body; and often it has proven a hand capable of reaching into the bounds of the spirit world, and of snatching from the jaws of death, patients asphyxiated with narcotic gases. It stimulates the muscles of respiration to contraction, carrying on the circulation until the noxious gases are thrown off; otherwise respiration would cease, and the heart's action would be stilled forever. Nelaton's method of producing artificial respiration is that most highly esteemed now, (here Nelaton's method was described) ; of course this would be tried in conjunction with the battery. The tongue must also be drawn forward to prevent its collapse closing the glottis.

Dr. Beach. "The paper is good, and discusses the subject from a scientific stand-point. This discussion is bringing out some points of a very practical value. I agree with Dr. Bartholow in regard to anæthesia. There is less danger of reflex action, more calmness and deliberation on the part of the operator, where the patient is fully anæsthetized. The effects of the oxygenated compounds, is due to an exalted vital action. I do not think they depress, even secondarily, the vital forces.

Dr. Noel. Would not the emersion of an animal in an atmosphere of carbonic acid, depress the vital processes, and finally stop them entirely? Does not carbonic acid arise in the blood from the action of these oxygenated anæsthetics, and is it not, in large quantity, necessarily depressing to the vital forces?

Dr. Prothro. Has any one experimented with the recent plan of producing anæsthesia by rapid breathing?

Dr. T. E. Beach. I have, in two or three cases, tried it with the happiest results, in one instance extracting two teeth with almost entire immunity from pain. I intend to continue to experiment with it. It costs nothing, is safe, and always on hand.

The subject of operative dentistry was now taken up. The Secretary opened the discussion by giving his method of filling some difficult cavities.

Dr. Ross. I generally fill the cervical portion of proximate cavities with non-cohesive, building up with cohesive gold. I now devitalize but few nerves. Fill all cavities that are deep and approach near the nerve, with oxychloride of zinc for a time, cutting away and finishing with gold.

Dr. Prothro. I have been anxious to get some new points about soft gold. I use cohesive gold entirely, and it consumes so much time that I can't get much done in a day. I fill front teeth generally from the front, separating, with the file or disk, before wedging there. Wedge quickly with hard wood. We should leave space. I do not, for this reason, like contour fillings. I leave a rounded shoulder, and take every precaution to prevent the approximation of the teeth. Use some amalgam, but have a predjudice against it for proximate fillings.

Dr. White. I believe in rapid separations, and wide ones, leaving a shoulder at the gum. I prefer soft gold at the cervical border ; generally build up half way with it, then cut a retaining pit, if necessary, or plant a screw to anchor the cohesive gold, with which I finish. There are some teeth that I cannot fill with gold; in such I use amalgam. Prefer a good amalgam filling, to an imperfect one of gold.

The doctor then gave his method of filling incisors on their proximate surfaces; which is, to leave a feather edge to the cavity at the incisive border.

Dr. Noel. "I am sorry to hear Dr. Prothro say he separates teeth so widely, and files before wedging. If we are going to wedge at all, let us do that first, and then mutilate the teeth as little with the file as possible.

Dr. Beach. There is much to be considered besides the mere method of filling teeth; but I shall confine myself to the subject in hand.

In cavities on the grinding surfaces, cut out freely fissures, if they are deep. Commence with non-cohensive gold cylinders at the distal portion of the fissure, taking lateral and anterior fissures successively, finishing in the centre of the cavity. In proximate surfaces of molar teeth, I separate with the disk, making a double shaped separation There is but one use for amalgam in dentistry, and that is, to fill such teeth as we cannot safely fill with gold. Oxychloride of zinc has been much abused as a capping fcr exposed nerves. I have had better success with gutta percha and other substances. A mixture of precip. chalk and creosote is less irritating to the pulp than oxide of zinc and creosote. Do not like gold for filling deciduous teeth. Frefer Hill's stopping or amalgam.

### AFTERNOON SESSION.

The name of Dr. S. M. Prothro, of Chattanooga, was presented by the Committee on Membership, and he was duly elected.

The regular business was now suspended, and the society proceeded to elect officers for the ensuing year, which resulted as follows: President, L. G. Noel, Nashville; First Vice-President, J. H. Webber, Springfield; Second Vice-president, S. M. Prothro, Chattanooga; Recording Secretary, H. E. Beach, Clarksville; Corresponding Secretary, J. C. Ross, Nashville; Treasurer, W. H. Morgan, Nashville. Executive Committee: S. J. Cobb, T. E. Beech, and W. H. Morgan. Nashville was chosen as the next place of meeting. The subject of operative dentistry was resumed.

Dr Noel gave his method of cleansing and filling the pulp canals of devitalized teeth. He condemned the practice of endeavoring to enlarge these canals with a drill. From their sinuosity it is often impossible to follow them, and your drill goes you know not where. Nature has left a smooth surface over which your filling will glide, touch it with a drill, and you mar it forever. If the canals are so small and sinuous you can't follow them, don't worry, fill the tooth with Hill's stopping, and when it gets sore take it out, repeat this until it will bear close stopping, then fill permanently, and *let the canals alone.*

Dr. Ross. "I have ceased to use gold to fill root canals, but fill them all with os artificial.

Dr. T. E. Beech. This is my method with one exception. I use a small pledget of cotton saturated in creosote, and rolled in oxide of zinc to carry up to the point of the root. I then fill with the oxychloride, using cotton on an instrument as a piston.

Dr. Lee. I have used the same method. My object in using the cotton at the apex, is to keep the oxychloride from going through.

Dr. H. E. Beach. In the operation of preparing and filling roots, I pursue a different course from any here mentioned. I extract the pulp as thoroughly as possible, and wipe out with cotton and creosote. I measure the length of my canals with a broach, passing it just through the apicial foramen. I then cut a piece of lead the length of my canal, dip in compound tincture of iodine, and drive it into the root.

The subject of pivoting teeth was next discussed, but no new points were brought out. Passing to the subject of dental prosthesis.

Dr. Beach. I am using celluloid. Think the plate I exhibited to the society last year was warped in finishing. I like the substance better every time I use it. Use dry heat—a little afraid of burning—think glycerine would be better. Use metalic models exclusively. Would like to use rubber but for the restrictions of the rubber company.

Dr. Ross. I have some trouble in vulcanite from the springing of plates, particularly if the arch is high.

Dr. Prothro. I have abandoned the use of air chambers entirely. Use wax nearly altogether for impressions.

Dr. Cook. I think Dr. Ross' trouble is not so much due to shrinkage, as to the fact that the centre of deep arches are softer and yield to pressure in taking impressions.

The subject of Etiology and Prophylaxis was now taken up.

Dr. Noel said he was in favor of filling sixth year molars as soon as decay commences, and wherever there is a prospect of crowding after the period of danger with these teeth is passed, he preferred to extract the first bicuspids on either side to give room for a spacing of the teeth. This course would always be found best where the canines lacked room to come in, and as a means of preventing proximate decay, was unsurpassed. Provides his patients with Codman's floss carries and silk floss. Thinks tooth-picks are frauds.

Dr. Beach related several cases illustrative of the heredity of carries.

Dr. Prothro. If we had reached an understanding of the causes of decay, we would have made a long stride. Cleanliness alone will not stop decay, but a neglect of cleanliness produces much disease. Malformation, want of lime salts, and want of cleanliness are three of the prime causes of decay. Think lime salts ought to be prescribed internally in many cases to furnish pabulum to the teeth.

Dr. Lee extended an invitation to the members of the State society to meet with the East Tennessee Dental Society, at Cleveland on the third Wednesday of September. Drs. Ross, L. C. Chisholm, and L. G. Noel, were appointed a committee to draft suitable resolutions relative to the death of Dr. Herman and the society ordered a memorial page to be left in the records for them.

A paper was now read upon the "Future of Dentistry," from the pen of Dr. Morgan, of Nashville. The paper deprecates, the practice of calling every practitioner of dentistry, doctor, regardless of the fact that neither the degree of D. D. S. or M. D. have been conferred upon the individual so styled. The discussion of this paper occupied the society up to the hour of adjournment. After conferring a vote of thanks upon Drs. Lee, Prothro, and Lincoln, of Chattanooga, and upon the proprietor of Lookout Mountain House for courtesies shown, the association adjourned.

